# Digitally Programmable Analogue Circuits for Sensor Conditioning Systems

**DOI:** 10.3390/s90503652

**Published:** 2009-05-14

**Authors:** Guillermo Zatorre, Nicolás Medrano, María Teresa Sanz, Concepción Aldea, Belén Calvo, Santiago Celma

**Affiliations:** 1 Teltronic S.A.U., R&D Department, Polígono Malpica, calle F-Oeste, 50057 Zaragoza, Spain; E-Mail: gzatorre@unizar.es; 2 Group of Electronic Design, Aragon Institute for Engineering Research, I3A, Facultad de Ciencias, Pedro Cerbuna 12, 50009 Zaragoza, Spain; E-Mails: caldea@unizar.es; becalvo@unizar.es; scelma@unizar.es; 3 Instituto Nacional de Astrofísica, Óptica y Electrónica, Luis Enrique Erro # 1, Tonantzintla, Puebla, Mexico; E-Mail: materesa@inaoep.mx (M.T.S.)

**Keywords:** sensor readout circuits, electronics for sensor conditioning, neural networks circuits

## Abstract

This work presents two current-mode integrated circuits designed for sensor signal preprocessing in embedded systems. The proposed circuits have been designed to provide good signal transfer and fulfill their function, while minimizing the load effects due to building complex conditioning architectures. The processing architecture based on the proposed building blocks can be reconfigured through digital programmability. Thus, sensor useful range can be expanded, changes in the sensor operation can be compensated for and furthermore, undesirable effects such as device mismatching and undesired physical magnitudes sensor sensibilities are reduced. The circuits were integrated using a 0.35 μm standard CMOS process. Experimental measurements, load effects and a study of two different tuning strategies are presented. From these results, system performance is tested in an application which entails extending the linear range of a magneto-resistive sensor. Circuit area, average power consumption and programmability features allow these circuits to be included in embedded sensing systems as a part of the analogue conditioning components.

## 1. Introduction

Recent technological advances allow a large number of battery-operated, inexpensive wireless networked sensor devices to be embedded in the physical environment. Wireless sensor networks (WSNs), allow device mobility, fast and easy installation and relocation according to needs. Application fields cover natural habitat monitoring, structure health controlling, environmental pollutants detection, seismic structural damage monitoring, industrial process control and military target tracking, among others [1].

A WSN unit typically contains a set of sensors monitoring physical variables. The processed values are transmitted by means of a radio transceiver working in an industrial-scientific-medical (ISM) band. The use of batteries to supply the system energy [1-2] permits some of their main features, such as mobility or system ubiquity. In order to achieve long battery life (months or even years), power consumption must be carefully managed.

A sensor unit can comprise smart sensors, with digital output and low power modes, and transducers that provide a raw analogue output. Interfacing between such sensors and the digital part of the system often requires conditioning electronics [3-4]. An interface circuit consists of an analogue section to improve the sensor output by extending its linear range and reducing cross-sensitivity to other physical variables, and analogue-digital converters (ADC) to digitize the data to be processed by a microcontroller. Programmability allows a more versatile operation for the interface circuit, which can change its behavior according to the requirements. A classical programmable solution is a polynomial compensation [5]. This solution can be affected by mismatches, reducing its performance. Currently we can see in the literature more sophisticated solutions, as in [6], where an analogue programmable circuit is presented to amplify the signal supplied by a sensor, compensating the output offset. In this case, the system merely fits the output signal span to the input range of the ADC available in the microcontroller, but the sensor non-linearities are not corrected. In [7], a versatile conditioning circuit for automotive applications is presented. In this case, the system consists of analogue and digital elements, and power is provided by the car battery, so the adaptation to portable battery operated applications is difficult.

This work presents two analogue cells intended to build sensor interfaces and signal conditioning circuits for portable applications. The interface response is digitally tuned, compensating non-linearities in the sensor response and undesired effects due to circuit components mismatching. The power consumption can be reduced by powering off the analogue components when the system is not sampling the sensor outputs. In this way, the battery life in portable systems is extended. The proposed circuits were integrated in a 0.35 μm standard digital CMOS process. The following sections show the use of current-mode analogue adaptive systems in sensor conditioning: design and characteristics of the proposed circuits, experimental measurements and loading effects. The feasibility of a complex conditioning architecture based on these cells is also demonstrated.

## 2. Adaptive Systems

Adaptive circuits in sensor conditioning permit tuning the circuit operation to match changes in sensor response due to ageing, environmental effects or sensor replacement, providing optimum performance under any condition by means of a tuning/calibration process. Perceptron [8-9] features make it a worthy candidate to be used in adaptive analogue-digital signal processing, where system operation is programmed by adjusting the values stored in a set of registers. Due to their robustness to circuit non-idealities, mismatches and offsets, tuning operation can be achieved by means of perturbative algorithms [10].

**Figure 1.**
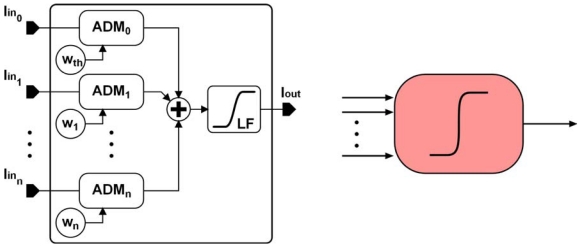
Proposed adaptive processing unit.

To embed sensor network units in a portable system, they must work with compact low-voltage batteries, making it difficult to process the data in voltage mode. Current-mode electronics give better results at low bias voltages [11]. The proposed processing elements were designed to provide good transfer features and impedance matching between them. The main current-mode circuits presented are a four-quadrant analogue-digital current multiplier (ADM) and a current amplifier that performs a logistic function. By properly combining both processing cells, it is possible to design a non-linear adaptive unit (Figure 1) which will be the basic cell in a multi-layer perceptron designed to extend the linear range of a sinusoidal sensor. [12-13].

## 3. Arithmetic Circuits

The conditioning circuit basically consists of two main blocks: an analog-digital current-mode four-quadrant multiplier (ADM) and a logistic circuit (LC) that performs a non-linear operation.

### 3.1. Four-Quadrant Multiplier

**Figure 2.**
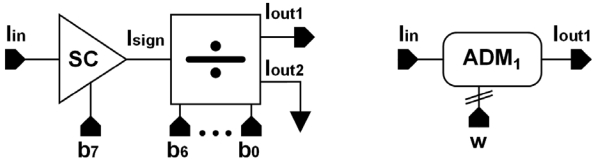
Four-quadrant analog-digital current multiplier.

The four-quadrant current-mode multiplier (Figure 2) is based on a MOS R-2R current ladder (M-2M ladder) [14], and a current follower as the sign circuit (SC). This circuit is a modified version of a cell that has been previously reported in the literature [15-16].

**Figure 3.**
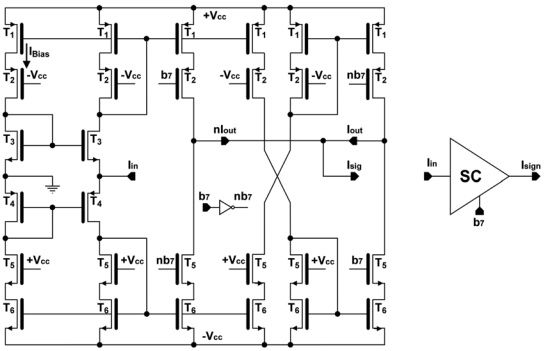
Sign circuit.

As shown in Figure 2, the most significant bit (b_7_) determines the direction of the current flow, that is, it selects the sign of the operation. The sign circuit is a current follower as shown in Figure 3.

Current mirrors are used to select the output either to I_out_ or nI_out_ according to the sign bit, while the non-selected output is set to high impedance. When b_7_ is ‘0’, the input current flows through the right-side output of the circuit (I_out_), keeping the same direction; when sign bit is ‘1’, the current flows through the left-side output (nI_out_) and its direction is reversed. The proposed SC provides to the output signal a symmetrical path independently of the value of the sign bit, improving the circuit output behavior compared to previous works [13], in which the signal path differs depending on the sign bit, altering the circuit operation. The output current from the sign circuit I_sign_ is driven to the M-2M ladder. Table 1 shows the main features of the circuit. As inferred from the input and output resistance values, the sign circuit not only reverses current direction if necessary, it also provides good impedance coupling to the next stage.

**Figure 4.**
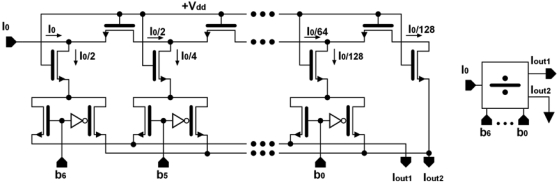
M-2M current ladder.

Figure 4 shows a 7-bit M-2M ladder. It is a classical circuit which divides the input current into two branches depending on the value of a set of programmed bits. In our case, Iout1 is the ladder output and Iout2 is grounded. The input current is multiplied by a factor Δ that depends on the value of the first 7-bits of an 8-bit register, according to:

(1) $$\Delta =\frac{1}{2^{n}}\left(\sum_{j=0}^{n-1}b_{j}2^{j}\right)\text{with n}=7$$

Table 2 summarizes the characteristics of this circuit. The complete operation is represented by:

(2) $$I_{\text{out}1}=wI_{in}\wedge w={\left(-1\right)}^{b_{7}}\frac{1}{2^{7}}\left(\sum_{j=0}^{6}b_{j}2^{j}\right)$$

### 3.2. Logistic Circuit

**Figure 5.**
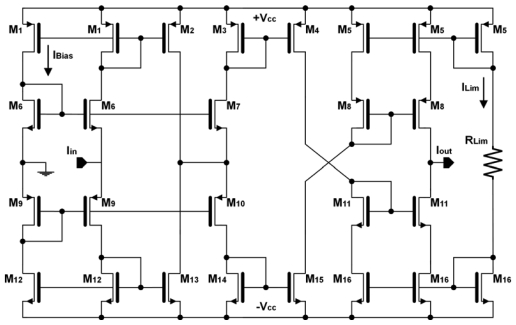
Non-linear (logistic) output circuit.

The logistic circuit shown in Figure 5 provides a non-linear output. It consists of a current amplifier with a bias current I_bias_=25 μA (for I_in_=0). Current I_Lim_ (right side of the schematic) limits the maximum absolute value of the output current to a predetermined value of 50 μA, providing the non-linear operation. In Table 3 the characteristics of the logistic circuit are shown. As in the previous cases, some characteristics depend on the input current.

## 4. Experimental Results

**Figure 6.**
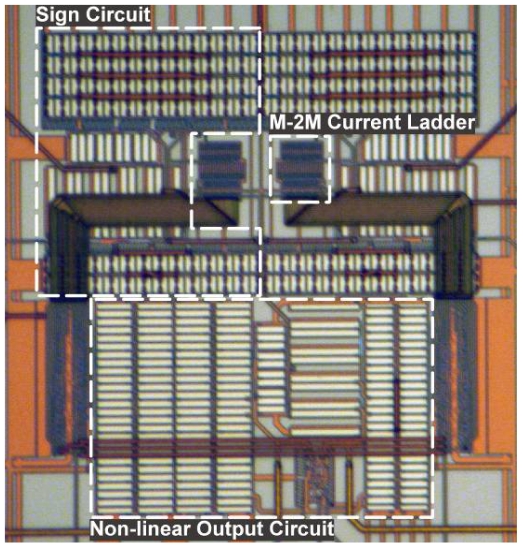
Chip microphotograph.

**Figure 7.**
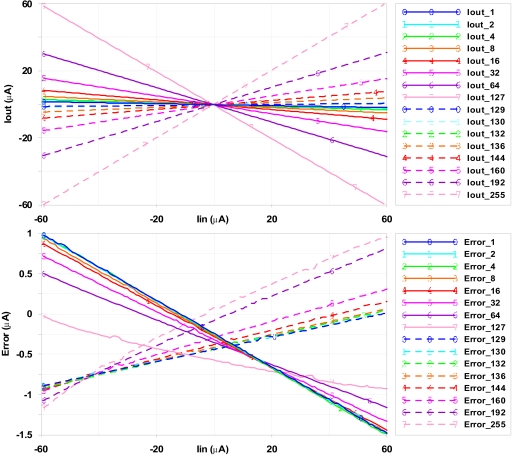
(Top) Current ladder output. (Bottom) Output error (compared to the expected output).

**Figure 8.**
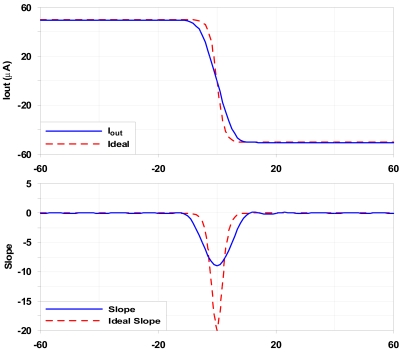

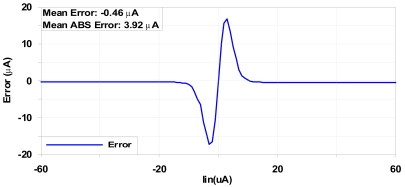
(Top) Logistic circuit operation compared to an ideal behaviour. (middle) Slope difference. (Bottom) Output error.

Prototypes of multipliers and logistic circuits were integrated in the 3.3 V–0.35 μm standard CMOS technology from *Austria Microsystems* (AMS). The corresponding layouts for these structures have been carefully realized taking into account matching between transistors and symmetry between sections. The main processing blocks are highlighted in the detail of the chip microphotography shown in Figure 6: sign circuit, M-2M ladder and logistic circuit. Maximum current is limited by the sign and logistic circuits. To provide more flexibility, both circuits were oversized so that they could be used in systems where higher currents are managed. The maximum output current of the logistic circuit can be increased or decreased by modifying the limiting resistor R_Lim_ (see Figure 5). Figures 7 and 8 represent experimental measurements of the aforementioned circuits. Experimental measurements accurately match post-layout simulation results [17].

**Figure 9.**
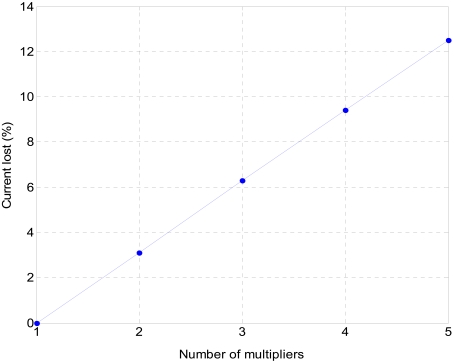
Current lost due to connecting several multipliers to a single logistic circuit (%).

By properly interconnecting the proposed analogue cells, it is possible to build adaptive analogue interfaces. Figure 1 shows the proposed adaptive processing unit. The logistic circuit input is connected to several multiplier cells. Depending on the number of multipliers connected to the non-linear circuit and the weight b_7_b_6_…b_0_ stored in the corresponding register, the current transfer to the logistic input changes, thus affecting the interface behavior. Post-layout simulations show that in the worst case (when multipliers present the minimum output impedance) the current transferred from a multiplier to a non-linear circuit decreases almost linearly by a mean value of about 3% per additional multiplier (Figure 9).

**Figure 10.**
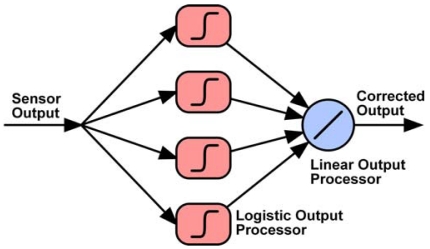
Sensor processing architecture.

Therefore, five multipliers connected to an output circuit, cause a mean error equal or lower than 12.5% in the current transfer. Deviations of the system behavior are compensated by the training algorithm, by properly fitting the weight values, thus preserving the system performance. Figure 10 shows a multi-layer perceptron based on the proposed processing units, which is designed to extend the linear range of a sinusoidal sensor. Processors in the middle layer have two inputs: the sensor output and an additional bias input (not shown in the figure). The processor in the output layer provides, as output, the weighted sum of outputs from the previous layer plus an additional bias current.

Post-layout and experimental results were taken into account to simulate a linearization circuit for angular position sensors. The architecture consists of a 1-4-1 multilayer perceptron (five weighted inputs in the output layer, including the bias input, similar to Figure 10). The goal of the conditioning circuit is to double the linear range of a giant magneto resistive (GMR) sensor, with a maximum tolerance of 1 degree in the angle estimation.

In order to program the operation of the conditioning circuit, we used algorithms based on parameter perturbation. These tuning techniques are less sensitive to processor non-idealities than gradient descent methods [18]. In [19], a study of the effects of mismatching in a previous version of

the proposed architecture was performed using Monte Carlo simulations, showing its capability to adaptation when the system is tuned using perturbative algorithms, even for relative errors greater than 30% in the operation of the circuits.

In this work, two different parameter perturbation strategies were tested: multiple-parameter and single-parameter perturbation. In each tuning strategy, the number of perturbed bits per parameter is fixed (from 1 to 7) and the number of iterations (perturbations) is limited to 400. Results are obtained by averaging 10 samples of training processes for each possible perturbation range and training strategy.

### 4.1. Training Algorithm: Multiple-Parameter Perturbation

**Figure 11.**
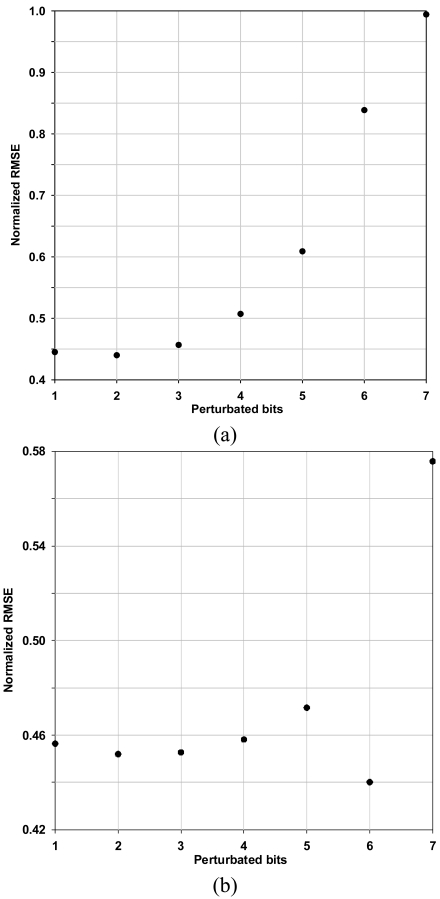
(a) RMSE vs. number of bits perturbed (multiple parameter algorithm). (b) RMSE vs. number of bits perturbed (single parameter algorithm).

In a Multiple-Parameter Perturbation, all the parameters of the conditioning circuit are modified in parallel and the root mean squared error (RMSE) of the output is calculated for the new parameter configuration. Only when the RMSE, calculated as the difference between the conditioned response and an ideal linear output, decreases, are the new values kept; otherwise, they are discarded and old data are kept. Figure 11a shows the final normalized RMSE as a function of the maximum number of bits that can be modified per parameter. We see that the minimum RMSE achieved after 400 iterations increases exponentially with the number of bits that can be modified, thus reducing the training performance.

### 4.2. Training Algorithm: Single-Parameter Perturbation

In a Single-Parameter Perturbation, the RMSE of the output is calculated after perturbing only one parameter. If the RMSE decreases, the new parameter value is kept; otherwise, it is discarded and the old value is kept. Figure 11b shows the final normalized RMSE. In this case, the error is almost constant even for perturbations of six bits per parameter.

The linear range of the sensor can be extended according to the specifications by means of either a multiple-parameter or a single-parameter algorithm. In both cases, the conditioning system achieves the expected performance. However, the single-parameter algorithm is a better choice for hardware implementation, as only a 1-to-6 bits perturbation is calculated per iteration, in contrast with the set of N 1-to-3 bits perturbations per iteration required for the multiple parameter algorithm applied to an N-weight conditioning circuit.

## 5. Conclusions

The paper presents two current-mode circuits designed as basic cells for sensor signal processing systems. They were implemented using a 0.35 μm standard digital CMOS technology, so that they could be easily integrated in the analogue core of a sensor interface ASIC. Digital programmability confers to the analogue circuitry the ability to compensate undesired effects as sensor output drifts, non-idealities or circuit mismatches, while keeping the conditioning features. Experimental results show accurate transfer functions, input and output impedances. In addition, what is remarkable is the relatively low sensitivity to effects due to the connection of several multipliers to a logistic circuit; however, if the number of elements to be connected were too high and the transfer error became difficult to compensate by tuning the system parameters, modifications in the processing architecture could be considered.

Results are presented of two perturbative algorithms (multiple and one-weight) applied to parameter tuning. It was shown that the single parameter perturbation algorithm provides better performance vs. hardware complexity ratio. In sensor network applications, the tuning process is controlled by the embedded low-cost processor that manages the node operation. Thus, updating time is mainly determined by the microprocessor operation and its clock frequency.

The proposed circuit shows a trade-off between size and power, providing good performance and signal transfer between basic building blocks. The use of perturbative learning compensates circuit non-idealities in multipliers and logistic circuits and current transfer lost, improving the full system operation. The use of the proposed circuits in battery-operated multi-sensor systems with low-frequency sampling, such as WSNs, can provide an analogue programmable pre-processing signal interface to the digital part of the sensing unit. Battery life can be extended by using intelligent power management, turning on (waking up) and off (sleeping) the corresponding electronics, keeping on only the power of the registers and turning off the analogue parts.

## Figures and Tables

**Table 1.** Sign circuit Characteristics.

**Active area**				13×10^-3^ mm^2^		
**Max. input resistance**				13 Ω		
**Mean input resistance**				9 Ω		
**Min. output resistance**				31 kΩ		
**Mean output resistance**				149 kΩ		
**Maximum power**				541 μW		
**Mean power**				457□μW		
**±V_cc_**				±1.65 V		
**b_7_, nb_7_**				±1.65 V		
**I_Bias_ (I_in_=0)**				30 μA		

**Transistors geometry**							

	**T1**	**T2**	**T3**	**T4**	**T5**	**T6**

**Width/Length (μm/μm)**	168/4	40/1	64/4	168/4	40/1	88/4

**Table 2.** Current ladder characteristics.

**Active area**	165.4 μm^2^
**Input resistance**	325 Ω
**Min. output resistance**	410 Ω
**Mean output resistance**	615 Ω
**Quiescent power**	79 pW
**+V_dd_**	1.65 V
**b_n_**	±1.65 V

**Transistors Geometry**	

**Width/Length (μm/μm)**	10/0.35

**Table 3.** Characteristics of the non-linear output circuit.

**Active area**				4.73×10^-3^ mm^2^				
**Max. input resistance**				13 Ω				
**Mean input resistance**				9 Ω				
**Min. output resistance**				12 kΩ				
**Mean output resistance**				15 kΩ				
**Maximum power**				2.0 mW				
**Mean power**				1.8 mW				

**Transistors geometry**								

	**M1**	**M2**	**M3**	**M4**	**M5**	**M6**	**M7**	**M8**

**Width/ Length (μm/μm)**	168/4	1680/4	8.5/1	17/1	6/1	64/4	3.1/1	3/1

	**M9**	**M10**	**M11**	**M12**	**M13**	**M14**	**M15**	**M16**

**Width/ Length (μm/μm)**	168/4	8.5/1	1/1	64/4	640/4	3.4/1	6.8/1	3/1
